# Health Care Utilization and Out-of-Pocket Expenses in the 30-, 60-, and 90-Day Postoperative Period After Hand Trauma

**DOI:** 10.1177/15589447251404983

**Published:** 2026-01-07

**Authors:** Jessica I. Billig, Yixin Tang, Michael Wu, Luyu Xie, Joshua M. Liao

**Affiliations:** 1 University of Texas Southwestern Medical Center, Dallas, USA

**Keywords:** health care utilization, postoperative, hand trauma, out-of-pocket expenses

## Abstract

**Background:**

There are knowledge gaps about postsurgical utilization and spending after hand trauma, which has implications for outcomes and access to care.

**Methods:**

Using 2019-2022 national claims, we examined 30-, 60-, and 90-day postsurgical health care utilization and out-of-pocket (OOP) expenses of flexor tendon repairs, open reduction internal fixation (ORIF) of a distal radius fracture, and digital replantation/revascularization. Multivariable regression models were used to examine the association between characteristics and postsurgical utilization and OOP expenses.

**Results:**

Among 22 170 patients, a large proportion of patients had utilization within 30 days (19 188; 87%), 60 days (20 022; 91%), and 90 days (20 170; 92%) after surgery, translating to OOP expenses of $41 at 30 days versus $107 at 90 days with substantial procedural variation (eg, 90-day OOP expenses after ORIF of a distal radius fracture of $98 vs $211 for flexor tendon repair). Patients undergoing flexor tendon repair had greater odds of reoperation/hospitalization at 30 days (adjusted odds ratio [aOR] = 1.8, 95% confidence interval [CI] = 1.5-2.1), 60 days (aOR = 1.8, 95% CI = 1.5-2.0), and 90 days (aOR = 1.6, 95% CI = 1.4-1.8) compared with ORIF of distal radius fractures. Flexor tendon repair was associated with 52% greater OOP expenses at 30 days (95% CI = 1.4-1.6) and 33% greater OOP expenses at 90 days (95% CI = 1.2-1.4) compared with ORIF of distal radius fractures.

**Conclusions:**

There was substantial variation in utilization and patient OOP expenses 30, 60, and 90 days postoperatively. These findings can help inform policy and practice leaders as they implement strategies to minimize patient financial burden of health care.

## Introduction

Hand trauma consists of a heterogeneous collection of injuries that account for about 42.3 million emergency department encounters annually.^1^ Surgical intervention for hand trauma can require differing levels of procedural complexity and postoperative recovery. As such, there are some data showing differing degrees of postsurgical utilization and associated spending for hand trauma.^2^ Understanding postoperative utilization is essential to improve patient outcomes and increase access to care. In addition, these data can be used to better define the postoperative course for patients after hand trauma, which may be used for future policy interventions. However, there are limited data regarding how postsurgical utilization varies at different time points during the expected postoperative period.

Moreover, there are limited data regarding how postoperative health care utilization translates to patient costs after hand trauma. As financial costs borne by the patient, out-of-pocket (OOP) expenses have been associated with worse therapeutic adherence and access to care.^3-6^ Yet, OOP expenses for medical care has been growing, and patients are bearing more of the burden of their health care utilization.^7-10^ Therefore, it is imperative to understand changes in OOP expenses after hand trauma over time.

Together, these knowledge gaps impede surgeons’ and surgical leaders’ ability to understand and address postsurgical care after hand trauma. This analysis of hand trauma and related surgery sought to address these knowledge gaps and inform efforts from policy and practice leaders to address postsurgical utilization and spending, which may be used to further define the postoperative episode for future policy interventions.

## Methods

### Data Source

We used 100% national health care claims from Merative Marketscan Commercial Claims and Encounters Database, which contains data from more than 23 million privately insured individuals in the United States.^11^ These databases contain de-identified longitudinal patient-level inpatient and outpatient encounters, health care costs, and other utilization measures including rehabilitation, durable medical equipment, and pharmaceutical claims. Due to the de-identified nature of the claims, this study qualified for exempt status from the institutional review board.

### Study Period and Cohort

Our study period spanned from January 1, 2018, to March 31, 2023. Our cohort included patients aged 18 years or older who underwent 1 of 3 procedures: open reduction internal fixation (ORIF) of distal radius fractures, flexor tendon repair, and revascularization/digital replantation in either the inpatient or outpatient setting. These surgical procedures were identified using Current Procedural Terminology (CPT) codes and International Classification of Diseases, 10th Revision (ICD-10) Procedure Codes (Appendix A) and included as index procedures in the analysis to provide a representative sample of hand trauma procedures that are health care resource intensive and commonly require postoperative utilization. Multiple procedures included any patient who had a combination of ORIF of a distal radius fracture, flexor tendon repair, and revascularization/digital replantation. The procedures were performed from January 1, 2019, to December 31, 2022. To capture 30-, 60-, and 90-day postoperative outcomes, we excluded patients who did not have continuous enrollment through 90 days after index procedures; to capture baseline patient comorbidities, we also excluded patients without continuous enrollment in the year preceding index procedures (Appendix B).

### Outcomes

Our primary outcomes were health care utilization in the 30-, 60-, and 90-day postoperative periods (yes/no), defined using ICD-10 diagnosis codes. To ensure that the postoperative health care utilization was associated with hand trauma, each health care utilization claim was captured using hand trauma-related ICD-10 codes (Appendix A). For inpatient procedures, the postoperative episode began after discharge and for the outpatient procedures, the postoperative episode began after the initial surgery. Among patients with any utilization, we categorized utilization as therapy, imaging/diagnostics, durable medical equipment/orthoses, hospitalizations, emergency department visits, office visits, postoperative visit during global period, post-acute care, reoperations (with anesthesia for the upper extremity as a surrogate to capture all reoperations), or other (eg, laboratory examinations). We then assessed reoperations and hospitalizations as its own outcome.

We captured copayment, coinsurance, and deductibles to calculate patient OOP expenses associated with postsurgical utilization (exclusive of OOP expenses related to index procedure). This OOP did not include the initial surgical intervention. Patients with claims with negative or zero payments and with negative OOP expenses for the entire episode (30, 60, or 90 days) were excluded, along with patients with claims in the lowest and highest 1% of payments and OOP expenses. Cost data were inflation-adjusted to 2022 dollar values.

### Characteristics

Characteristics used in the analysis included patient age, sex, geographic location, per capita income based on metropolitan statistical area for 2022, insurance type, procedure type, and patient health status (determined by Charlson Comorbidity Index, CCI, using ICD-10 codes in the 1 year before the index surgery).^12,13^

### Statistical Analysis

We conducted descriptive analyses to compare patient characteristics with and without utilization in the 30-, 60-, and 90-day postoperative period. We then calculated the OOP expenses associated with the utilization during each time period (30, 60, and 90 days) and used Kruskal-Wallis test to examine the difference among OOP at 30, 60, and 90 days postoperatively.

Multivariable logistic regression was used to examine the association between characteristics and any reoperations/hospitalization. Covariates in this model included age, sex, geographic region, insurance type, CCI score, initial operative setting, and procedure type. Generalized linear models with a log link and gamma distribution were used to examine the association between characteristics and patient OOP expenses. In this model, we used patients with OOP expenses greater than zero. In this model, the regression coefficients were exponentiated to obtain cost ratios. Covariates in the model included age, sex, geographic region, insurance type, CCI score, initial operative setting (inpatient vs outpatient), procedure type, reoperation, or hospitalization. We used 2-tailed statistical tests with alpha set at 0.05.

## Results

Our cohort included 22 170 patients. Of these, 19 188 (87.1%) had health care utilization in the 30-day postoperative period, 20 022 (90.9%) in the 60-day postoperative period, and 20 170 (91.6%) in the 90-day postoperative period. Within the 90-day cohort, 86% of the patients (17 259 patients) underwent ORIF of a distal radius fracture, 12% (2355 patients) underwent flexor tendon repairs, 2% (341 patients) underwent revascularization/digital replantation, and 1% (215 patients) underwent multiple procedures.

Comparing patients with and without any postsurgical health care utilization (Appendix C), patients with utilization were older compared with patients without utilization in the 30-, 60-, and 90-day postoperative period. Any utilization was more common among patients undergoing ORIF of distal radius fractures (92% of patients 30 days; 96% of patients at 60 days; 96% of patients at 90 days) and flexor tendon repairs (76% of patients at 30 days; 80% of patients at 60 days; 82% of patients at 90 days) compared with digital replantation/revascularization.

**Figure 1.**
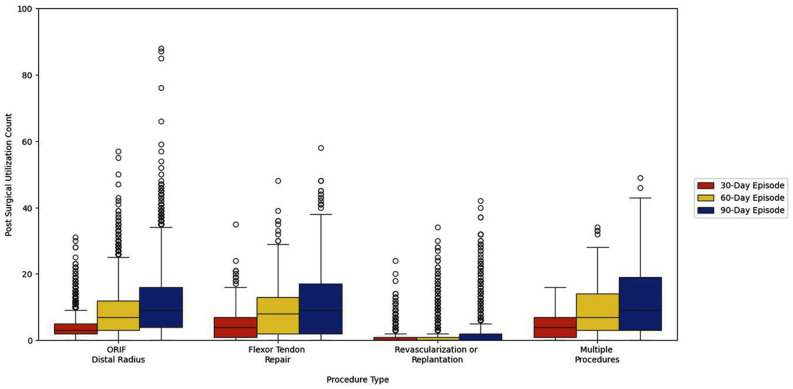
Box plot of postsurgical utilization count stratified by procedure type. ORIF = open reduction internal fixation.

Among patients with any health care utilization, no differences were observed in patient characteristics (age, sex, per capita income) comparing across 30-, 60-, and 90-day postoperative period (Table 1). However, patients undergoing ORIF of distal radius fracture had greater extent of utilization in the 90-day postoperative compared with the 30- and 60-day postoperative period (Figure 1). The median OOP expenses for patients was $41 (quartile 1-quartile 3: $0-$139) at 30 days, $81 (quartile 1-quartile 3: $0-$262) at 60 days, and $107 (quartile 1-quartile 3: $2-$347) at 90 days (Table 1).

**Table 1.** Comparison of Postoperative Health Care Utilization in the 30-, 60-, and 90-Day Period After Hand Trauma (N = 22 034).

Patient characteristics	30-day (N = 19 188)	60-day (N = 20 022)	90-day (N = 20 170)
Age			
18-34	3452 (17.99%)	3633 (18.15%)	3677 (18.23%)
35-44	2587 (13.48%)	2698 (13.48%)	2723 (13.50%)
45-54	4478 (23.34%)	4668 (23.31%)	4714 (23.37%)
55-64	8671 (45.19%)	9023 (45.07%)	9056 (44.90%)
Sex			
Female	12 954 (67.51%)	13 459 (67.22%)	13 534 (67.10%)
Male	6234 (32.49%)	6563 (32.78%)	6636 (32.90%)
MSA per capita personal income			
Below 25th percentile (<$55 477)	2424 (12.63%)	2549 (12.73%)	2565 (12.72%)
25th to 75th percentile ($55 477-$64 292)	4914 (25.61%)	5094 (25.44%)	5124 (25.40%)
Above 75th percentile (>$64 292)	2422 (12.62%)	2522 (12.60%)	2540 (12.59%)
Unknown	9428 (49.13%)	9857 (49.23%)	9941 (49.29%)
Geographic region			
North Central	4402 (22.94%)	4564 (22.79%)	4590 (22.76%)
Northeast	2946 (15.35%)	3055 (15.26%)	3073 (15.24%)
South	8601 (44.82%)	9005 (44.98%)	9081 (45.02%)
West	3239 (16.88%)	3398 (16.97%)	3426 (16.99%)
Insurance type			
PPO	9406 (49.02%)	9849 (49.19%)	9933 (49.25%)
HMO	2193 (11.43%)	2323 (11.60%)	2338 (11.59%)
High deductible	5210 (27.15%)	5401 (26.98%)	5436 (26.95%)
Other	2379 (12.40%)	2449 (12.23%)	2463 (12.21%)
CCI score			
Zero	13 322 (69.43%)	13 882 (69.33%)	13 982 (69.32%)
One or greater	5866 (30.57%)	6140 (30.67%)	6188 (30.68%)
Setting			
Outpatient	17 703 (92.26%)	18 366 (91.73%)	18 469 (91.57%)
Inpatient	1485 (7.74%)	1656 (8.27%)	1701 (8.43%)
Procedure type			
Distal radius fracture treatment	16 531 (86.15%)	17 177 (85.79%)	17 259 (85.57%)
Flexor tendon repair	2198 (11.46%)	2315 (11.56%)	2355 (11.68%)
Digital replantation/revascularization	265 (1.38%)	318 (1.59%)	341 (1.69%)
Multiple procedures	194 (1.01%)	212 (1.06%)	215 (1.07%)
Post surgery utilization			
Total post surgery utilization	14 491 (75.52%)	16 579 (82.80%)	16 883 (83.70%)
Imaging and diagnostics	13 180 (68.69%)	13 732 (68.58%)	13 895 (68.89%)
DME/orthoses	10 622 (55.36%)	14 307 (71.46%)	15 054 (74.64%)
Therapy	2659 (13.86%)	3640 (18.18%)	4226 (20.95%)
Other	1667 (8.69%)	2406 (12.02%)	3030 (15.02%)
Office visit	1104 (5.75%)	1286 (6.42%)	1339 (6.64%)
Postoperative visit during global period	946 (4.93%)	1294 (6.46%)	1558 (7.72%)
Reoperation	320 (1.67%)	333 (1.66%)	341 (1.69%)
Hospitalization	174 (0.91%)	207 (1.03%)	231 (1.15%)
Emergency department visit	45 (0.23%)	50 (0.25%)	51 (0.25%)
Post acute care	3452 (17.99%)	3633 (18.15%)	3677 (18.23%)
Median OOP (Quartile 1-Quartile 3)	41.32 (0-138.93)	81.24 (0-262.39)	107.15 (1.98-347.25)

CCI = Charlson Comorbidity Index; DME = durable medical equipment; HMO = health maintenance organization; MSA = metropolitan statistical area; OOP = out-of-pocket; PPO = preferred provider organization.

Figure 1 summarizes utilization at 30, 60, and 90 days postoperatively, stratified by procedure type. Utilization varied substantially by procedure. However, there was increasing utilization from 30 to 90 days for all procedures. This variation in utilization corresponded to differences in median OOP expenses comparing 30, 60, and 90 days (ORIF of distal radius fracture: $37 [quartile 1-quartile 3: $0-$123] at 30 days vs $98 [quartile 1-quartile 3: $0-$325] at 90 days, *P* < .001; flexor tendon repairs: $105 [quartile 1-quartile 3: $16-$246] at 30 days vs $211 [quartile 1-quartile 3: $31-$499] at 90 days, *P* < .001, Table 2).

**Table 2.** Median Out-of-Pocket Expenses at 30, 60, and 90 Days Postoperatively Stratified by Procedure Type.

Procedure type	Median 30-day OOP (Q1-Q3)	Median 60-day OOP (Q1-Q3)	Median 90-day OOP (Q1-Q3)	*P*-value*
ORIF of distal radius	36.57 (0-123.05)	73.8 (0-243.92)	97.99 (0-325.43)	.000
Flexor tendon repair	104.63 (15.54-245.50)	172.49 (22.96-406.40)	211.24 (30.59-499.28)	.000
Revascularization/replantation	21.89 (0-140.0)	30.71 (0-202.62)	34.97 (0-247.33)	.141
Multiple procedures	72.21 (0-247.33)	100.55 (0-387.14)	118.87 (0-488.04)	.084

OOP = out-of-pocket; ORIF = open reduction internal fixation; Q1 = quartile 1; Q3 = quartile 3.

* *P*-value obtained by Kruskal-Wallis test.

In multivariable analyses, we observed variation in patient characteristics associated with reoperation/hospitalization at 30, 60, and 90 days postoperatively. At 30 days, patients aged 45 to 54 had a 17% lower odds of having a postoperative reoperation/hospitalization compared with patients aged 55 to 64 (adjusted odds ratio [aOR] = 0.83, 95% confidence interval [CI] = 0.71-0.97, Table 3). There was a similar association at 60 days postoperatively, but not at 90 days postoperatively. We also found geographic variation (differences in utilization in North Central and Northeast compared with the South) at all time points.

**Table 3.** Logistic Regression Examining the Association of Patient Characteristics and Reoperation/Hospitalization at 30-, 60-, and 90-Day Postoperative Period.

Patient characteristics	30-day OR (95% CI)	60-day OR (95% CI)	90-day OR (95% CI)
Age			
18-34	0.83 (0.7-0.98)	0.82 (0.71-0.96)	0.84 (0.73-0.97)
35-44	0.79 (0.65-0.95)	0.77 (0.65-0.91)	0.84 (0.72-0.98)
45-54	0.83 (0.71-0.97)	0.84 (0.74-0.97)	0.91 (0.8-1.03)
55-64	1 (reference)		
Sex			
Female	1 (reference)		
Male	1.23 (1.08-1.4)	1.33 (1.19-1.49)	1.45 (1.3-1.61)
Geographic region			
North Central	0.61 (0.52-0.71)	0.72 (0.63-0.83)	0.72 (0.64-0.82)
Northeast	0.49 (0.4-0.6)	0.53 (0.44-0.63)	0.55 (0.47-0.65)
South	1 (reference)		
West	0.73 (0.62-0.86)	0.81 (0.7-0.94)	0.8 (0.69-0.92)
Insurance type			
PPO	1 (reference)		
HMO	0.94 (0.78-1.14)	0.99 (0.84-1.17)	0.99 (0.85-1.16)
High deductible	0.86 (0.75-1)	0.87 (0.77-0.99)	0.92 (0.82-1.04)
Other	0.91 (0.75-1.11)	1.07 (0.91-1.27)	1.03 (0.88-1.21)
CCI score			
Zero	1 (reference)		
One or greater	1.17 (1.03-1.32)	1.14 (1.02-1.28)	1.12 (1.01-1.24)
Setting			
Outpatient	1 (reference)		
Inpatient	1.51 (1.26-1.81)	1.91 (1.64-2.22)	2.09 (1.82-2.4)
Procedure type			
Distal radius fracture	1 (reference)		
Flexor tendon repair	1.8 (1.54-2.1)	1.76 (1.53-2.02)	1.58 (1.38-1.8)
Replantation/revascularization	0.62 (0.45-0.87)	0.54 (0.41-0.73)	0.48 (0.37-0.64)
Multiple procedures	2.39 (1.6-3.55)	2.36 (1.66-3.36)	1.99 (1.42-2.8)

CCI = Charlson Comorbidity Index; HMO = health maintenance organization; OR = odds ratio; PPO = preferred provider organization; CI = confidence interval.

There were also substantial differences in postoperative reoperation/hospitalization outcomes between different procedure types. Patients undergoing flexor tendon repairs had a 1.8 times greater odds of reoperation/hospitalization at 30 days (aOR = 1.8, 95% CI = 1.54-2.1), 1.76 times increased odds at 60 days (aOR = 1.76, 95% CI = 1.53-2.02), and 1.58 times increased odds at 90 days (aOR = 1.58, 95% CI = 1.38-1.80) compared with patients undergoing ORIF of distal radius fractures (Table 3). Patients undergoing revascularization/digital replantation had lower odds of reoperation/hospitalization at 30, 60, and 90 days postoperatively compared with patients undergoing ORIF of a distal radius fracture (Table 3).

**Figure 2.**
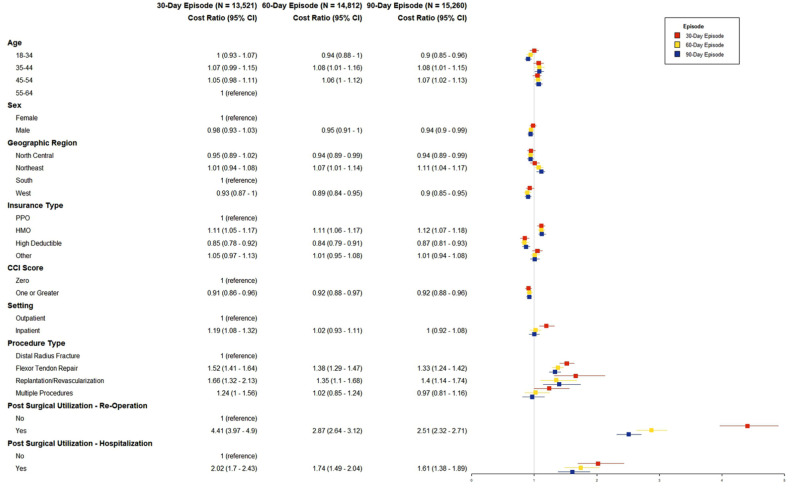
Postsurgical out-of-pocket expenses at 30-, 60-, and 90-day episodes. CCI = Charlson Comorbidity Index; CDHP = consumer-directed health plan; HDHP = high-deductible health plan; HMO = health maintenance organization; PPO = preferred provider organization; POS = point of service.

Figure 2 summarizes characteristics associated with patient OOP expenses. We found that increasing age was associated with higher OOP. We also observed differences in the association between procedural type and patient OOP expenses. Compared with those undergoing ORIF of distal radius fractures, flexor tendon repair was associated with 52% greater OOP expenses at 30 days (95% CI = 1.41-1.64), 38% greater OOP expenses at 60 days (95% CI = 1.29-1.47), and 33% greater OOP expenses at 90 days (95% CI = 1.24-1.42, Figure 2). However, there were no differences in digital replantation/revascularization compared with ORIF of distal radius fractures. In addition, we found differences in OOP if patients had postoperative reoperations or hospitalizations at all 3 time points compared with patients who did not have reoperations or hospitalizations postoperatively.

## Discussion

In this nationwide study of patients undergoing surgery after hand trauma, we characterize the postoperative health care utilization and associated OOP expenses at 30, 60, and 90 days postoperatively. Our findings highlight the differences in utilization based on procedure type with a larger percentage of patients with flexor tendon injuries having utilization during the initial 30-day postoperative period compared with a larger percentage of patients with ORIF of distal radius fractures with sustained utilization throughout the 90-day postoperative period. This translates to differing OOP expenses, which are stable for ORIF of distal radius fractures from 30 to 90 days and doubles for flexor tendon repairs from 30 to 90 days postoperatively. These findings offer new insights and build on prior work in several ways that can inform future changes in surgical care.

First, this study yields new information about postoperative health care utilization at 30, 60, and 90 days after common hand trauma procedures. Previous work has shown that complexity of hand injury is associated with longer hospital lengths of stay and reoperation rates.^14,15^ However, there is little information on the overall health care utilization after hand trauma procedures at different time period and how specific procedures affect this utilization. We found that postoperative health care utilization increased steadily for patients undergoing ORIF of distal radius fractures from 30 to 90 days, but not for those undergoing other procedures (eg, most utilization after flexor tendon repairs and digital replantation/revascularization occurred during the 30-day postoperative period rather than further at 60 or 90 days postoperatively, but was higher than ORIF of distal radius fractures at all time points).

For less complex non-hand surgery procedures, most of the utilization and associated spending can be predicted in the 30-day postoperative period^2^; but that for more complex procedures requiring longer hospitalizations and more post-acute care, 30-day utilization does not predict the 90-day utilization.^16-19^ Many hand trauma procedures can be performed in the outpatient setting, thus making them “less complex,” but we found continued utilization for specific procedures (ie, flexor tendon repairs) beyond the 30-day window. This may be due to the need for long postoperative rehabilitation in the form of hand therapy, which may contribute to the procedural heterogeneity of our utilization findings. Our findings indicate that a uniform global period may not fit all hand trauma procedures and potentially the length of the global period should be stratified by procedure type.

Second, we filled knowledge gaps by characterizing how health care utilization at 30, 60, and 90 days postoperatively after hand trauma translates to patient OOP expenses. Recent data have shown growing patient OOP expenses.^7-10^ In our study, we find increasing OOP expenses in the 30-, 60-, and 90-day postoperative period that varies by procedure type. Some procedures, such as ORIF of distal radius fractures, have very little change over the 90 days, compared with flexor tendon repairs where OOP expenses doubled from 30 to 90 days postoperatively. Previous work has focused on changes in OOP expenses over the years or OOP expenses by site of service, yet there is little information regarding how OOP expenses change during different time points during the global period.^7,9,20^ This information is important because patients rarely understand their OOP expenses before utilizing health care services—especially after emergent/urgent surgery when certain aspects of postoperative care are bundled into the global period and others are not. These data can help provide a national estimate of how much patients have to pay for their postoperative care after hand trauma and can help inform shared decision-making discussions between providers and patients.

Given the heterogeneity of patient OOP expenses, researchers have pushed for episode-based cost sharing to promote high value care without penalizing those with complications or more complex postoperative courses.^21^ Moreover, every insurance plan is different with varying copayments, coinsurance, and deductibles, thus muddying price transparency after surgery. Despite recent legislation mandating hospital price transparency, there continues to be low compliance, commercial prices for the same procedure vary widely across and within hospitals, and price transparency rules do not include non-bundled postoperative services, such as hand therapy.^22-25^ Therefore, a better understanding of the postoperative OOP expenses is necessary to provide patients with their financial obligations for their postoperative care after hand trauma.

This study has limitations. First, because of inherent limitations in claims data, the analysis lacked granular clinical data that may affect postoperative health care utilization after hand trauma, such as injury severity or surgeon skill. Second, this study assessed health care utilization and patients OOP expenses to address knowledge gaps across a policy- and practice-salient time window; future work should assess even long-term complications and patient costs. Third, use of commercial claims may limit generalizability of findings to patients with other types of insurance, such as Medicaid or Medicare.

Nonetheless, this nationwide analysis reveals insights about utilization and patient OOP expenses after hand trauma and surgery. Beyond filling knowledge gaps about hand trauma surgery, these findings can help inform surgeons and surgical leaders as they continue to implement strategies to improve surgical care and minimize patient financial burden of health care delivery.

## Supplemental Material

sj-docx-1-han-10.1177_15589447251404983 – Supplemental material for Health Care Utilization and Out-of-Pocket Expenses in the 30-, 60-, and 90-Day Postoperative Period After Hand Trauma Supplemental material, sj-docx-1-han-10.1177_15589447251404983 for Health Care Utilization and Out-of-Pocket Expenses in the 30-, 60-, and 90-Day Postoperative Period After Hand Trauma by Jessica I. Billig, Yixin Tang, Michael Wu, Luyu Xie and Joshua M. Liao in HAND

sj-docx-3-han-10.1177_15589447251404983 – Supplemental material for Health Care Utilization and Out-of-Pocket Expenses in the 30-, 60-, and 90-Day Postoperative Period After Hand Trauma Supplemental material, sj-docx-3-han-10.1177_15589447251404983 for Health Care Utilization and Out-of-Pocket Expenses in the 30-, 60-, and 90-Day Postoperative Period After Hand Trauma by Jessica I. Billig, Yixin Tang, Michael Wu, Luyu Xie and Joshua M. Liao in HAND

sj-pptx-2-han-10.1177_15589447251404983 – Supplemental material for Health Care Utilization and Out-of-Pocket Expenses in the 30-, 60-, and 90-Day Postoperative Period After Hand Trauma Supplemental material, sj-pptx-2-han-10.1177_15589447251404983 for Health Care Utilization and Out-of-Pocket Expenses in the 30-, 60-, and 90-Day Postoperative Period After Hand Trauma by Jessica I. Billig, Yixin Tang, Michael Wu, Luyu Xie and Joshua M. Liao in HAND
