# Supplementary material for: Health Care Utilization and Out-of-Pocket Expenses in the 30-, 60-, and 90-Day Postoperative Period After Hand Trauma
Source: Hand (N Y). 2026 Jan 7:15589447251404983. Online ahead of print. doi: 10.1177/15589447251404983 (PMC12783034; doi:10.1177/15589447251404983)
Supplement: sj-docx-1-han-10.1177_15589447251404983 – Supplemental material for Health Care Utilization and Out-of-Pocket Expenses in the 30-, 60-, and 90-Day Postoperative Period After Hand Trauma [file sj-docx-1-han-10.1177_15589447251404983.docx]

Appendix A: CPT/ICD-10 Codes Used for 30-, 60-, and 90-Day Postoperative Period Construction

| **CPT Codes for Surgical Procedure** | |
| --- | --- |
| ORIF of Distal Radius Fracture | 25607, 25608, 25609 |
| Flexor Tendon Repair | 26350, 26352, 26356, 26357, 26358, 26370, 26372, 26373 |
| Digital Replantation/Revascularization | 20816, 20822, 20824, 20827, 35206, 35207, 35236, 35266 |
| **ICD-10 Procedure Codes for Surgery** | |
| ORIF of Distal Radius Fracture | 0PSHX, 0PSJ0X |
| Flexor Tendon Repair | 0LM7X, 0LM8X, 0LQ5X, 0LQ6X, 0LQ7X, 0LQ8X |
| Digital Replantation/Revascularization | 0XMLX, 0XMMX, 0XMNX, 0XMPX, 0XMQX, 0XMRX, 0XMSX, 0XMTX, 0XMVX, 0XMWX, 03QDX, 03QFX |
| **ICD-10 Codes for Utilization** | |
| Distal radius fracture | S52.5X, S62.101D, S62.101G, S62.102G, S62.102D |
| Injury of flexor tendon | S56.0X, S56.1X, S56.2X, S66.0X, S66.1X |
| Hand/Wrist Pain | M79.601, M79.602, M79.603, M79.62, M79.63, M79.64, M25.53X |
| Finger Nonunion/Malunion | S62.201K, S62.201P, S62.202K, S62.202P, S62.209K, S62.202P, S62.251K, S62.251P, S62.252K, S62.252P, S62.253K, S62.253P, S622.291K, S62.291P, S62.292K, S62.292P, S62.299K, S62.299P, S62.300K, S62.300P, S62.301K, S62.301P, S62.302K, S62.302P, S62.303K, S62.303P, S62.304K, S62.304P, S62.305K, S62.305P, S62.306K, S62.306P, S62.307K, S62.307P, S62.308K, S62.308P, S62.309K, S62.309P, S62.310K, S62.310P, S62.311K, S62.311P, S62.312K, S62.312P, S62.313K, S62.313P, S62.314K, S62.314P, S62.315K, S62.315P, S62.316K, S62.316P, S62.317K, S62.317P, S62.318K, S62.318P, S62.319K, S62.319P, S62.320K, S62.320P, S62.321K, S62.322P, S62.323K, S62.323P, S62.324K, S62.324P, S62.325K, S62.325P, S62.326K, S62.326P, S62.327K, S62.327P, S62.328K, S62.328P, S62.329K, S62.329P, S62.330K, S62.330P, S62.331K, S52.331P, S62.332P, S62.332K, S62.333K, S62.333P, S62.334K, S62.334P, S62.335K, S62.335P, S62.336K, S62.336P, S62.337K, S62.337P, S62.338K, S62.338P, S62.339K, S62.339P, S62.340K, S62.390K, S62.390P, S62.391K, S62.391P, S62.392K, S62.392P, S62.393K, S62.393P, S62.394K, S62.394P, S62.395K, S62.395P, S62.396K, S62.396P, S62.397K, S62.397P, S62.398K, S62.398P, S62.399K, S62.399P, S62.501K, S62.501P, S62.502K, S62.502P, S62.509K, S62.509P, S62.511K, S62.511P, S62.512K, S62.512P, S62.513K, S62.513P, S62.521K, S62.521P, S62.522K, S62.522P, S62.523K, S62.523P, S62.600K, S62.600P, S62.601K, S62.601P, S62.602K, S62.602P, S62.603K, S62.603P, S62.604K, S62.604P, S62.605K, S62.605P, S62.606K, S62.606P, S62.607K, S62.607P, S62.608K, S62.608P, S62.609K, S62.609P, S62.610K, S62.610P, S62.611K, S62.611P, S62.612K, S62.612P, S62.613K, S62.613P, S62.614K, S62.614P, S62.615K, S62.615P, S62.616K, S62.616P, S62.617K, S62.617P, S62.618K, S62.618P, S62.619K, S62.619P, S62.620K, S62.620P, S62.621K, S62.621P, S62.622K, S62.622P, S62.623K, S62.623P, S62.624K, S62.624P, S62.625K, S62.625P, S62.626K, S62.626P, S62.627K, S62.627P, S62.628K, S62.628P, S62.629K, S62.629P, S62.630K, S62.630P, S62.631K, S62.631P, S62.632K, S62.632P, S62.633K, S62.633P, S62.634K, S62.634P, S62.635K, S62.635P, S62.636K, S62.636P, S62.637K, S62.637P, S62.638K, S62.638P, S62.639K, S62.639P |
| Hand/finger/wrist stiffness | M25.63X, M25.64X |
| Tendon rupture | M66.20, M66.23X, M66.24X, M66.28, M66.30, M66.33X, M66.34X, M66.83X, M66.84X, S66.811, S66.812, S66.819, S66.911, S66.912, S66.919 |
| Tendon Adhesions | M67.83X, M67.84X, M67.93X, M67.94X |
| Orthopedic Aftercare | Z47.81, Z47.89 |
| Removal of Hardware | Z47.2, Z98.890 |
| Neuroma | T87.0X, T87.31, T87.32 |
| Infection/Osteomyelitis | M00.00, M00.03X, M00.04X, M00.10, M00.13X, M00.14X, M00.20, M00.23X, M00.24X, M00.80, M00.83X, M00.84X, M65.03X, M65.04X, M65.13X, M65.14X, M86.03X, M86.04X, M86.10, M86.13X, M86.14X, M86.23X, M86.24X, M86.33X, M86.34X, M86.43X, M86.44X, M86.53, M86.54, M86.63X, M86.64X, M86.8X3, M86.8X4, M86.8X9, T87.41, T87.42 |
| Wound | S61.0X, S61.1X, S61.2X, S61.3X, S61.4X, S61.5X, T87.51, T87.52, T87.8X, Z87.828 |
| Finger/Thumb Amputation | S68.X |
| Status post ORIF | Z98.890, Z87.81 |
| **CPT/HCPCS Codes for Utilization** | |
| Hospitalization | 99217, 99218, 99219, 99220, 99224, 99225, 99226, 99221, 99222, 99223, 99231, 99232, 99233, 99234, 99235, 99236, 99238, 99239, 99251, 99252, 99253, 99254, 99255 |
| Emergency Department Visit | 99281, 99282, 99283, 99284, 99285 |
| Clinic Visit | 99201, 99202, 99203, 99204, 99205, 99211, 99212, 99213, 99214, 99215, 99241, 99242, 99243, 99244, 99245, 99441, 99442, 99443, 99024, 99341, 99342, 99343, 99344, 99345, 99347, 99348, 99349, 99350 |
| Imaging and diagnostics | 73070, 73080, 73090, 73100, 73110, 73115, 73120, 73130, 73140, 73200, 73201, 73202, 73206, 73218, 73219, 73220, 73221, 73222, 73223, 73225, 76000, 76881, 76882 |
| Post Acute Care | 99304, 99305, 99306, 99307, 99308, 99309, 99310, 99315, 99316 |
| Anesthesia for the Upper Extremity (Surrogate for re-operation) | 01810, 01820, 01829, 01830, 01832, 01840, 01842, 01844, 01850, 01860, 01991, 64415, 64416, 64417 |
| Therapy | 97001, 97003, 97002, 97004, 97039, 97028, 97022, 90901, 98960, 97010, 97012, 97014, 97018, 97024, 97026, 97032, 97033, 97034, 97035, 97036, 97110, 97140, 97124, 97139, 97140, 97760, 97035, 97530, 97535, 97545, 97033, 97110, 97161, 97162, 97163, 97164, 97165, 97166, 97167, 97168, 97760, 97762, 97799, 97810, 97811, 97813, 97814, G0157, G0158, G0159, G0160 |
| DME/Orthoses | E0720, E0730, E0731, E1802, E1805, E1806, L3763, L3764, L3765, L3766, L3806, L3807, L3808, L3809, L3900, L3901, L3904, L3905, L3906, L3908, L3912, L3913, L3915, L3916, L3917, L3918, L3919, L3921, L3923, L3924, L3925, L3927, L3929, L3930, L3931, L3933, L3935, L3956, L3982, L3984, L3995, L3999, L4205, Q4005, Q4006, Q4009, Q4010, Q4013, Q4014, Q4017, Q4018, Q4021, Q4022, Q4050, Q4051 |

***X means include all of the numbers, usually 0-9.

CPT: current procedural terminology; HCPCS: healthcare common procedure coding system; ICD: International Classification of Disease
