# Supplementary material for: Health Care Utilization and Out-of-Pocket Expenses in the 30-, 60-, and 90-Day Postoperative Period After Hand Trauma
Source: Hand (N Y). 2026 Jan 7:15589447251404983. Online ahead of print. doi: 10.1177/15589447251404983 (PMC12783034; doi:10.1177/15589447251404983)
Supplement: sj-docx-3-han-10.1177_15589447251404983 – Supplemental material for Health Care Utilization and Out-of-Pocket Expenses in the 30-, 60-, and 90-Day Postoperative Period After Hand Trauma [file sj-docx-3-han-10.1177_15589447251404983.docx]

Appendix C: Comparison of Patient Characteristics With and Without Healthcare Utilization during the 30-, 60-, and 90-day Postoperative Period

| **30-Day** | | | |
| --- | --- | --- | --- |
| **Patient Characteristics** | **With Utilization (N=19,188)** | **Without Utilization**  **(N= 2,836)** | **P-Value** |
| Age |  |  |  |
| 18-34 | 3,452 (17.99%) | 730 (25.74%) | <0.001 |
| 35-44 | 2,587 (13.48%) | 462 (16.29%) | <0.001 |
| 45-54 | 4,478 (23.34%) | 660 (23.27%) | <0.001 |
| 55-64 | 8,671 (45.19%) | 984 (34.70%) | <0.001 |
| Sex |  |  |  |
| Female | 12,954 (67.51%) | 1,458 (51.41%) | <0.001 |
| Male | 6,234 (32.49%) | 1,378 (48.59%) | <0.001 |
| MSA Per Capita Personal Income |  |  |  |
| Below 25th percentile (< $55,477) | 2,370 (12.35%) | 402 (14.17%) | <0.001 |
| 25th to 75th percentile ($55,477-$64,292) | 4,968 (25.89%) | 648 (22.85%) | <0.001 |
| Above 75th percentile (> $64,292) | 2,422 (12.62%) | 320 (11.28%) | <0.001 |
| Unknown | 9,428 (49.13%) | 1,466 (51.69%) | <0.001 |
| Geographic Region |  |  |  |
| North Central | 4,402 (22.94%) | 592 (20.87%) | 0.027 |
| Northeast | 2,946 (15.35%) | 412 (14.53%) | 0.027 |
| South | 8,601 (44.82%) | 1,337 (47.14%) | 0.027 |
| West | 3,239 (16.88%) | 495 (17.45%) | 0.027 |
| Insurance Type |  |  |  |
| PPO | 9,406 (49.02%) | 1,514 (53.39%) | <0.001 |
| HMO | 2,193 (11.43%) | 368 (12.98%) | <0.001 |
| High Deductible | 5,210 (27.15%) | 690 (24.33%) | <0.001 |
| Other | 2,379 (12.40%) | 264 (9.31%) | <0.001 |
| CCI Score |  |  |  |
| Zero | 13,322 (69.43%) | 1,754 (61.85%) | <0.001 |
| One or Greater | 5,866 (30.57%) | 1,082 (38.15%) | <0.001 |
| Setting |  |  |  |
| Outpatient | 17,703 (92.26%) | 1,969 (69.43%) | <0.001 |
| Inpatient | 1,485 (7.74%) | 867 (30.57%) | <0.001 |
| Procedure Type |  |  |  |
| Distal Radius Fracture Treatment | 16,531 (86.15%) | 1,364 (48.10%) | <0.001 |
| Flexor Tendon Repair | 2,198 (11.46%) | 690 (24.33%) | <0.001 |
| Digital Replantation/Revascularization | 265 (1.38%) | 735 (25.92%) | <0.001 |
| Multiple Procedures | 194 (1.01%) | 47 (1.66%) | <0.001 |
| **60-Day** | | | |
| **Patient Characteristics** | **With Utilization (N=20,022)** | **Without Utilization (N=2,002)** | **P-Value** |
| Age |  |  |  |
| 18-34 | 3,633 (18.15%) | 549 (27.42%) | <0.001 |
| 35-44 | 2,698 (13.48%) | 351 (17.53%) | <0.001 |
| 45-54 | 4,668 (23.31%) | 470 (23.48%) | <0.001 |
| 55-64 | 9,023 (45.07%) | 632 (31.57%) | <0.001 |
| Sex |  |  |  |
| Female | 13,459 (67.22%) | 953 (47.60%) | <0.001 |
| Male | 6,563 (32.78%) | 1,049 (52.40%) | <0.001 |
| MSA Per Capita Personal Income |  |  |  |
| Below 25th percentile | 2,490 (12.44%) | 282 (14.09%) | 0.002 |
| 25th to 75th percentile | 5,153 (25.74%) | 463 (23.13%) | 0.002 |
| Above 75th percentile | 2,522 (12.60%) | 220 (10.99%) | 0.002 |
| Unknown | 9,857 (49.23%) | 1,037 (51.80%) | 0.002 |
| Geographic Region |  |  |  |
| North Central | 4,564 (22.79%) | 430 (21.48%) | 0.472 |
| Northeast | 3,055 (15.26%) | 303 (15.13%) | 0.472 |
| South | 9,005 (44.98%) | 933 (46.60%) | 0.472 |
| West | 3,398 (16.97%) | 336 (16.78%) | 0.472 |
| Insurance Type |  |  |  |
| PPO | 9,849 (49.19%) | 1,071 (53.50%) | <0.001 |
| HMO | 2,323 (11.60%) | 238 (11.89%) | <0.001 |
| High Deductible | 5,401 (26.98%) | 499 (24.93%) | <0.001 |
| Other | 2,449 (12.23%) | 194 (9.69%) | <0.001 |
| CCI Score |  |  |  |
| Zero | 13,882 (69.33%) | 1,194 (59.64%) | <0.001 |
| One or Greater | 6,140 (30.67%) | 808 (40.36%) | <0.001 |
| Setting |  |  |  |
| Outpatient | 18,366 (91.73%) | 1,306 (65.23%) | <0.001 |
| Inpatient | 1,656 (8.27%) | 696 (34.77%) | <0.001 |
| Procedure Type |  |  |  |
| Distal Radius Fracture Treatment | 17,177 (85.79%) | 718 (35.86%) | <0.001 |
| Flexor Tendon Repair | 2,315 (11.56%) | 573 (28.62%) | <0.001 |
| Digital Replantation/Revascularization | 318 (1.59%) | 682 (34.07%) | <0.001 |
| Multiple Procedures | 212 (1.06%) | 29 (1.45%) | <0.001 |
| **90-Day** | | | |
| **Patient Characteristics** | **With Utilization (N=20,170)** | **Without Utilization (N=1,854)** | **P-Value** |
| Age |  |  |  |
| 18-34 | 3,677 (18.23%) | 505 (27.24%) | <0.001 |
| 35-44 | 2,723 (13.50%) | 326 (17.58%) | <0.001 |
| 45-54 | 4,714 (23.37%) | 424 (22.87%) | <0.001 |
| 55-64 | 9,056 (44.90%) | 599 (32.31%) | <0.001 |
| Sex |  |  |  |
| Female | 13,534 (67.10%) | 878 (47.36%) | <0.001 |
| Male | 6,636 (32.90%) | 976 (52.64%) | <0.001 |
| MSA Per Capita Personal Income |  |  |  |
| Below 25th percentile | 2,504 (12.41%) | 268 (14.46%) | 0.002 |
| 25th to 75th percentile | 5,185 (25.71%) | 431 (23.25%) | 0.002 |
| Above 75th percentile | 2,540 (12.59%) | 202 (10.90%) | 0.002 |
| Unknown | 9,941 (49.29%) | 953 (51.40%) | 0.002 |
| Geographic Region |  |  |  |
| North Central | 4,590 (22.76%) | 404 (21.79%) | 0.705 |
| Northeast | 3,073 (15.24%) | 285 (15.37%) | 0.705 |
| South | 9,081 (45.02%) | 857 (46.22%) | 0.705 |
| West | 3,426 (16.99%) | 308 (16.61%) | 0.705 |
| Insurance Type |  |  |  |
| PPO | 9,933 (49.25%) | 987 (53.24%) | <0.001 |
| HMO | 2,338 (11.59%) | 223 (12.03%) | <0.001 |
| High Deductible | 5,436 (26.95%) | 464 (25.03%) | <0.001 |
| Other | 2,463 (12.21%) | 180 (9.71%) | <0.001 |
| CCI Score |  |  |  |
| Zero | 13,982 (69.32%) | 1,094 (59.01%) | <0.001 |
| One or Greater | 6,188 (30.68%) | 760 (40.99%) | <0.001 |
| Setting |  |  |  |
| Outpatient | 18,469 (91.57%) | 1,203 (64.89%) | <0.001 |
| Inpatient | 1,701 (8.43%) | 651 (35.11%) | <0.001 |
| Procedure Type |  |  |  |
| Distal Radius Fracture Treatment | 17,259 (85.57%) | 636 (34.30%) | <0.001 |
| Flexor Tendon Repair | 2,355 (11.68%) | 533 (28.75%) | <0.001 |
| Digital Replantation/Revascularization | 341 (1.69%) | 659 (35.54%) | <0.001 |
| Multiple Procedures | 215 (1.07%) | 26 (1.40%) | <0.001 |

CCI: Charlson comorbidity index; HMO: health maintenance organization; MSA: metropolitan statistical area; PPO: preferred provider organization
