# Supplementary material for: Health Care Utilization and Out-of-Pocket Expenses in the 30-, 60-, and 90-Day Postoperative Period After Hand Trauma
Source: Hand (N Y). 2026 Jan 7:15589447251404983. Online ahead of print. doi: 10.1177/15589447251404983 (PMC12783034; doi:10.1177/15589447251404983)
Supplement: sj-pptx-2-han-10.1177_15589447251404983 – Supplemental material for Health Care Utilization and Out-of-Pocket Expenses in the 30-, 60-, and 90-Day Postoperative Period After Hand Trauma [file sj-pptx-2-han-10.1177_15589447251404983.pptx]

## Slide 1
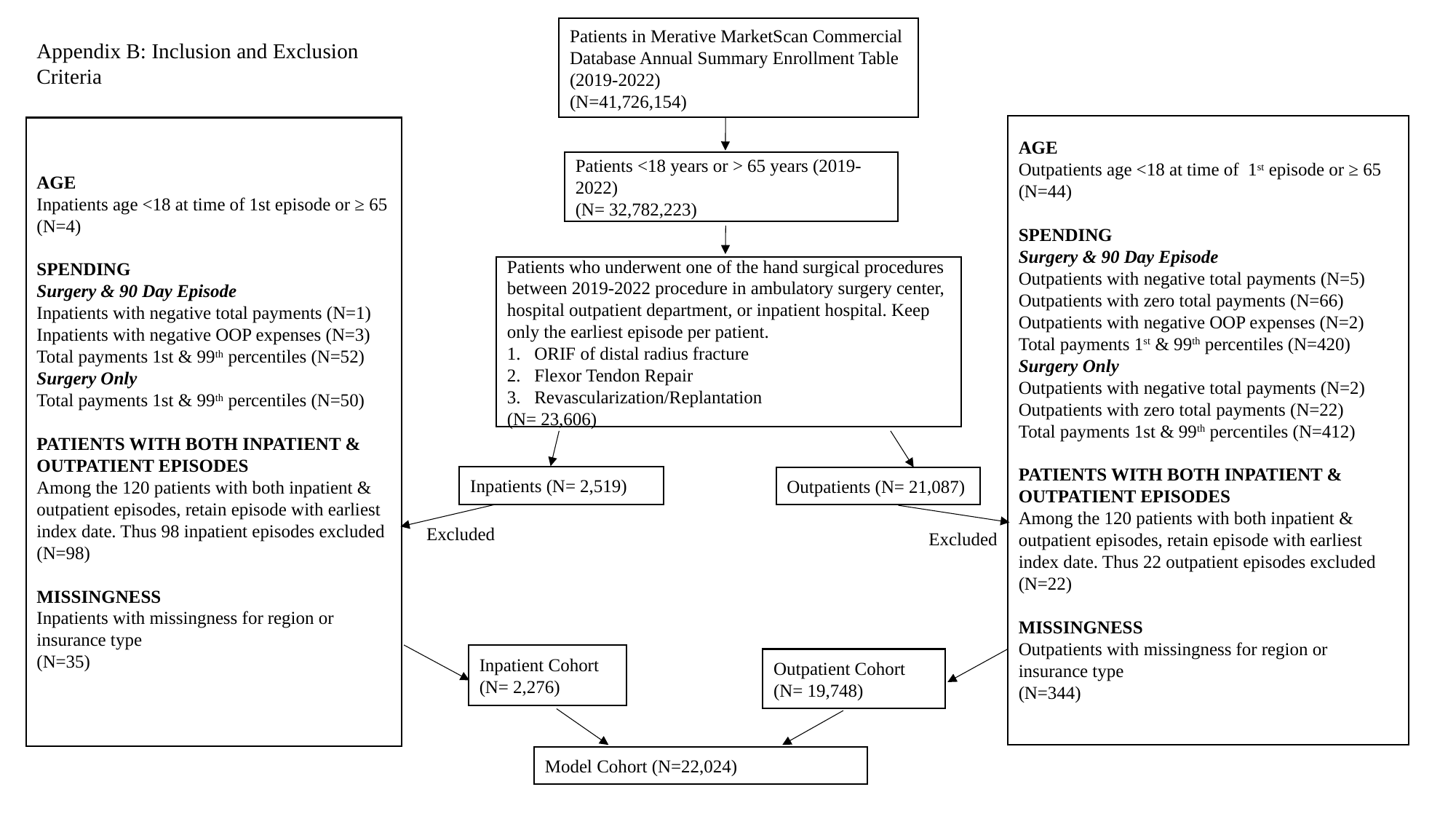

Patients in Merative MarketScan Commercial Database Annual Summary Enrollment Table (2019-2022)
(N=41,726,154)
Appendix B: Inclusion and Exclusion Criteria
AGE
Outpatients age <18 at time of 1st episode or ≥ 65 (N=44)
SPENDING
Surgery & 90 Day Episode
Outpatients with negative total payments (N=5)
Outpatients with zero total payments (N=66)
Outpatients with negative OOP expenses (N=2)
Total payments 1st & 99th percentiles (N=420)
Surgery Only
Outpatients with negative total payments (N=2)
Outpatients with zero total payments (N=22)
Total payments 1st & 99th percentiles (N=412)
PATIENTS WITH BOTH INPATIENT & OUTPATIENT EPISODES
Among the 120 patients with both inpatient & outpatient episodes, retain episode with earliest index date. Thus 22 outpatient episodes excluded (N=22)
MISSINGNESS
Outpatients with missingness for region or insurance type
(N=344)
AGE
Inpatients age <18 at time of 1st episode or ≥ 65 (N=4)
SPENDING
Surgery & 90 Day Episode
Inpatients with negative total payments (N=1)
Inpatients with negative OOP expenses (N=3)
Total payments 1st & 99th percentiles (N=52)
Surgery Only
Total payments 1st & 99th percentiles (N=50)
PATIENTS WITH BOTH INPATIENT & OUTPATIENT EPISODES
Among the 120 patients with both inpatient & outpatient episodes, retain episode with earliest index date. Thus 98 inpatient episodes excluded (N=98)
MISSINGNESS
Inpatients with missingness for region or insurance type
(N=35)
Patients <18 years or > 65 years (2019-2022)
(N= 32,782,223)
Patients who underwent one of the hand surgical procedures between 2019-2022 procedure in ambulatory surgery center, hospital outpatient department, or inpatient hospital. Keep only the earliest episode per patient.
ORIF of distal radius fracture
Flexor Tendon Repair
Revascularization/Replantation
(N= 23,606)
Inpatients (N= 2,519)
Outpatients (N= 21,087)
Excluded
Excluded
Inpatient Cohort (N= 2,276)
Outpatient Cohort (N= 19,748)
Model Cohort (N=22,024)
